# Scope and Predictors of Travel Medicine Practice among Primary Care Physicians in Qatar

**DOI:** 10.1016/j.pmedr.2023.102337

**Published:** 2023-07-22

**Authors:** Ayman Al-Dahshan, Nagah Selim, Noora Al-Kubaisi, Ziyad Mahfoud, Vahe Kehyayan

**Affiliations:** a Department of Medical Education, Community Medicine Residency Program, Hamad Medical Corporation, Doha, Qatar; b Department of Public Health and Preventive Medicine, Cairo University Kasr Alainy Faculty of Medicine, Cairo, Egypt; c Department of Clinical Affairs, Primary Health Care Corporation, Doha, Qatar; d Department of Global and Public Health, Weill Cornell Medical College, Doha, Qatar; e Department of Healthcare Management, University of Doha for Science & Technology, Doha, Qatar

**Keywords:** Travel medicine, Practice, Physicians, Primary healthcare, Qatar

## Abstract

In an era of globalization, travel-related illnesses have become a focus of public health concern. Pretravel consultation is an effective measure to promote healthy travel. This study aimed to assess the scope of primary care physicians’ (PCPs) practice of travel medicine (TM) in Qatar and its associated predictors. This was a cross-sectional study design. A structured questionnaire was used to collect data from all PCPs working in the 27 primary healthcare centers in Qatar. Descriptive and analytic statistics were used as appropriate, and a multivariable logistic regression model was constructed. Three hundred sixty-four PCPs participated in the study (response rate of 89.2%). Most PCPs (91.1%) provided pretravel consultations of which 72.7% provided less than 10 consultations per month. Overall, pretravel advice content and frequency including vaccine and malaria chemoprophylaxis recommendations were inadequate. Significant predictors of high frequency of pretravel consultations (≥10/month) included male PCPs (AOR 1.78, 95% CI: 1.01, 3.18), PCPs who had postgraduate training or experience in TM (AOR 2.74, 95% CI: 1.59, 4.72), and multilingual (speaking ≥3 languages) physicians (AOR 1.96, 95% CI: 1.12, 3.45). Frequently encountered post-travel illnesses included travelers’ diarrhea, respiratory diseases, and fever. While, most PCPs provided pretravel consultations, the frequency and content of consultations were inadequate. Male PCPs, past training or experience in TM, and multilingual physicians were important predictors of providing a high frequency of pretravel consultation. The findings of this study identified several gaps in PCPs’ TM practice. Specific measures should be designed and implemented to reduce the burden of travel-related illnesses and promote healthy travel.

## 1 Introduction

The primary concern of travel medicine (TM) is the prevention and management of health-related risks and conditions associated with travel (Aba et al., 2019). Globally, international travel has been increasing substantially reaching 1.5 billion in 2019 (United Nations World Tourism Organization. Growth in international tourist arrivals continues to outpace the economy., 2020). Qatar has witnessed a rapid development in the number of travelers with about 2.1 million arrivals in 2019, an increase of 17% compared to 2018 (Qatar National Tourism Council, 2018). Its strategy has been to establish itself as a unique destination for tourism; job opportunities, and world-class sports events. The overall impact of these changes will result in a massive growth in the two-way flow of travelers for business, leisure, and transit purposes (Qatar National Tourism Council, 2018, Frost & Sullivan & Insights Middle East, 2019).

Globalization, affordability of travel, and borderless countries across the continent have led to substantial increases in travel for either business or tourism (United Nations World Tourism Organization. International Tourism Results, 2017). Associated with increased travel, the risk of travel-related illnesses has also increased; for instance, 43% to 79% of travelers who visited developing countries became ill (Grieve and Steffen, 2020). Travel-related illness has become a primary concern for public health authorities (United Nations World Tourism Organization. International Tourism Results, 2017). This is particularly of greater concern in the Arab world where primary care is fragmented and travel-related health services are inadequate and not well-established (United Nations Sustainable Developmental Group, xxxx).

Pretravel consultation is an effective intervention in preventing travel-related illnesses and promoting travelers’ health (Sanford et al., 2016). It offers travelers a unique opportunity to prepare themselves for any health risks that might arise during their travels and to mitigate these risks (Gherardin, 2007). Therefore, it is vital for primary care physicians (PCPs) to provide all travelers with pretravel consultations tailored to their planned destinations (Abu Salem et al., 2015). They are best suited to practice TM because of their broad scope of training and their skills in counseling and disease prevention. (Abu Salem et al., 2015). Topics that are discussed in any pretravel consultation are widely varied based on the differences in travelers’ characteristics, planned travel destinations, or planned activities. But conventionally they should include advice regarding vaccine-preventable diseases, avoidance of animal and insect bites, malaria chemoprophylaxis, information on food and water-borne diseases, safety measures, guidelines for self-treatment of common illness during travel, and concluding with travel medical insurance (Aw et al., 2014).

Several worldwide studies have investigated the practice of TM among PCPs since the late 1980s. A study conducted by Kogelman et al. (Kogelman et al., 2014) in the United States found that more than two-thirds of the PCPs provided pretravel consultation. However, most of them saw only a small number of travelers (less than 50/year) (Kogelman et al., 2014). Another nationwide study in Germany showed that about two-thirds of PCPs saw at least 10 travelers per month with immunizations being the most common practice (95%) in their pretravel consultations, followed by advice on malaria chemoprophylaxis (94%), and risky exposure prophylaxis (41%) (Ropers et al., 2004). Few studies related to TM have also been conducted in the Arab region. For instance, a study conducted in Oman found only 58% of the PCPs provided pretravel consultations (Kurup et al., 2019).

In Qatar, TM services including travel vaccinations and malaria chemoprophylaxis are mainly offered by PCPs practising in Primary Health Care (PHC) centers (Corporation and Care, 2018). However, there is scant knowledge about the scope of their TM practice and associated factors. Therefore, the purpose of this study was 1) to assess the frequency and content of pretravel advice and associated predictors of providing a higher frequency of pretravel advice among PCPs, and 2) to assess the frequency of counseling specific patient groups about pretravel advice and the frequency of travel-related illnesses in returned travelers.

## 2 Materials and methods

### 2.1 Study design and setting

An analytical, descriptive cross-sectional design was used for this study. The study was conducted from March to May 2020 at all 27 PHC centers in Qatar. Primary health care centers are considered the first line of contact with Qatar’s healthcare system for the provision of a broad range of health services including TM services (e.g., travel vaccines and malaria chemoprophylaxis) (Corporation and Care, 2018). These services are offered by PCPs and are provided free of charge to Qatari citizens and at highly subsidized rates to expatriates.

### 2.2 Study population

All 408 PCPs who were on duty at the 27 PHC centers during the study period were invited to participate in the study. Those who were not available at the time (n = 44) of data collection (e.g., were on leave or in quarantine due to COVID-19) were excluded. Assuming a response rate of approximately 80%, the study with 326 responders will be able to estimate the prevalence of pretravel consultation within a margin of error of at most ± 5.5% using a 95% confidence interval. This assumption is based on a prevalence of about 50%, which is a conservative estimate that yields the largest margin of error. Additionally, this sample size will be sufficiently large enough to create a logistic regression model with at least 6 variables following the rule of thumb proposed by Peduzzi et al. (Peduzzi et al., 1996) and validated by Bujang et al. (Bujang et al., 2018). Each individual PCP, even if belonging to the same PHC center, was considered independent from each other as all PHC centers receive standardized guidelines for treating patients.

### 2.3 Data collection

The lead investigator (AAD) approached all eligible PCPs individually in their respective clinics and provided a fulsome information about the nature and scope of the study, and invited them to participate. To ensure privacy and confidentiality, the questionnaire used in the study was fully anonymized, with no personal identifiers collected from participants. Those who consented to participate were given a copy of the questionnaire and instructed to keep their completed questionnaires in sealed and unmarked envelopes. Designated data collectors collected the envelopes at the end of the day.

The present study assessed PCPs’ practice of TM in the preceding 6-month period (i.e., prior to the data collection date).

### 2.4 Study questionnaire

A self-administered, structured questionnaire was used for data collection. To ensure the content and face validity of the questionnaire, we followed two steps. First, we conducted an extensive search of the relevant literature. Second, we formed an expert panel of TM experts and Community Medicine consultants to critically review it. The questionnaire was in English because it is the main communication language of all physicians in Qatar. The development of the questionnaire also considered its suitability for self-administration. A pilot study was conducted with a convenient sample of 20 PCPs to assess its relevance, clarity, and average duration for its completion. The pilot sample was excluded from the final database. The estimated average time to complete the questionnaire was 18 min.

The questionnaire is composed of four sections: (i) background and general practice characteristics of PCPs including their age, gender, nationality, number of years in general practice, language(s) spoken with patients, postgraduate experience or training in TM, frequency of pretravel consultations per month, average duration of pretravel consultations; (ii) content and frequency of pretravel advice; (iii) frequency of counseling specific traveler patient groups; and (iv) frequency of travel-related illnesses encountered. For the purpose of this study, postgraduate experience in TM was defined as *“any engagement in travel medicine practice after graduation from medical school”*, and postgraduate training in TM was *“defined as receiving any postgraduate degree [Diploma, Master, PhD] or training [workshop, certified short course] or having a membership or fellowship of TM related professional organization“*.

### 2.5 Disclosure of ethical compliance

The study was conducted in compliance with the guidelines of the Primary Health Care Corporation (PHCC) and Hamad Medical Corporation (HMC) for the protection of human subjects concerning safety and privacy. Approval was obtained from the ethics committee of the HMC [Reference number: MRC-01–19-324] and the PHCC [Reference number: PHCC/DCR/2020/01/002].

### 2.6 Statistical analysis

Data collected from completed questionnaires were analysed using *IBM SPSS Statistics for Windows* (version 23, IBM Corp., Armonk, N.Y., USA). All variables were summarized using frequency distributions for categorical variables, and medians and interquartile ranges (IQR) for numerical variables. Univariable and multivariable logistic regression analyses were performed to identify predictors of high frequency (≥10) pretravel consultations per month. Unadjusted and adjusted odds ratios with their 95% confidence intervals (CI) and p-values were also reported. Statistical significance was considered at a two-sided p ≤ 0.05.

## 3 Results

### 3.1 Background characteristics of the study population

A total of 364 PCPs completed and returned the questionnaires giving a response rate of 89.2%. The median (IQR) age of the PCPs was 43 (39–49) years. Participants’ nationalities included British (41.7%), Egyptian (19.1%), Qataris (6.2%), and Sudanese (6.2%). About one-third (31.2%) spoke with patients in three or more languages. More than half (59.1%) had 15 years or more of experience in general practice. Less than half (42.8%) of PCPS had postgraduate training or experience in TM. The PCPs’ background characteristics are shown in Table 1.

**Table 1:** Distribution of primary care physicians’ background characteristics and their association with the frequency of pretravel consultations per month, 2020 (N = 364).

Variable	Total, n (%)^b^ (n = 364)	Frequency of TM consultation (n = 332)^c^	
		<10/month, n (%)	≥10/month, n (%)
Age			
<45 years old	205 (59.6)	145 (75.5)	47 (24.5)
≥45 years old	139 (40.4)	86 (74.1)	30 (25.9)
Median (IQR)	43 (39–49)		
Gender			
Male	215 (59.1)	135 (68.5)	62 (31.5)
Female	149 (40.9)	107 (82.3)	23 (17.7)
Nationality (n = 324)			
British	135 (41.7)	92 (76.0)	29 (24.0)
Egyptian	62 (19.1)	42 (73.7)	15 (26.3)
Qatari	20 (6.2)	16 (88.9)	2 (11.1)
Sudanese	20 (6.2)	12 (66.7)	6 (33.3)
Jordanian	16 (4.9)	8 (66.7)	4 (33.3)
Pakistani	13 (4.0)	7 (63.6)	4 (36.4)
Iraqi	11 (3.4)	9 (90.0)	1 (10.0)
Others^a^	47 (14.5)	–	–
Number of spoken languages with patients			
1-2 languages	249 (68.8)	172 (77.5)	50 (22.5)
≥3 languages	113 (31.2)	68 (66.0)	35 (34.0)
Median (IQR)	2 (2–3)		
Number of years in practice			
<15 years	146 (40.9)	104 (80.0)	26 (20.0)
≥15 years	211 (59.1)	133 (69.6)	58 (30.4)
Median (IQR)	18 (13–23)		
Postgraduate training or experience in TM			
No	206 (57.2)	145 (81.5)	33 (18.5)
Yes	154 (42.8)	95 (65.1)	51 (34.9)
Number of patients seen by PCPs per day			
<20 patients	57 (15.7)	38 (77.6)	11 (22.4)
20-29 patients	239 (65.7)	167 (78.0)	47 (22.0)
≥30 patients	68 (18.6)	36 (57.1)	27 (42.9)
Frequency of pretravel consultation per month (n = 332)^c^			
<10 consultations	242 (74.0)	_	_
10-19 consultations	58 (17.7)	_	_
≥20 consultations	27 (8.3)	_	_
Average duration of pretravel consultation (n = 332)^c^			
<10 min	141 (42.5)	_	_
10-19 min	162 (48.8)	_	_
≥20 min	29 (8.7)	_	_

a include: Indian, American, Australian, Syrian, Tunis and Philippines;

b column percentage;

c PCPs who provided pretravel consultation.

Overall, 91.1% of PCPs provided pretravel consultations to travelers in the six months preceding the data collection (95% CI: 88.3%-94.1%). Among these PCPs (n = 332; 91.1%), about three-quarters (74.0%) counseled less than 10 patients monthly on TM-related issues. The remaining PCPs advised 10 to 19 (17.7%), and 20 or more (8.3%) travelers per month.

Regarding the average duration of pretravel consultations, almost half (48.8%) of PCPs spent between 10 and 19 min per consultation. The remaining PCPs either spent less than 10 min (42.5%) or 20 min or more (8.7%) for each consultation.

### 3.2 Pretravel advice: Content and Frequency

Table 2 describes the frequency of counseling and type of advice to travelers according to their destination. Among PCPs who offered pretravel consultations (N = 332), the more frequently (i.e., *every time and often*) reported advice given was about safe water and food (51.1% every time and 33.8% often), traveler’s diarrhea (37.8% every time and 40.5% often), insect bite avoidance (38.5% every time and 39.4% often), and in-flight exercise to prevent deep vein thrombosis (DVT) (36.5% every time and 38.9% often). The less frequently reported advice given was about travel health insurance (13.1% every time and 23.8% often), first aid knowledge (17.6% every time and 28.9% often), Jet lag (19.5% every time and 28.0% often), and risk and prevention of Sexual Transmitted Infections (STIs) (19.0% every time and 32.4% often).

**Table 2:** Distribution of counseling travelers (according to specific destinations) about pretravel advice in the preceding 6 months in Qatar, 2020 (N = 332).

Variable	Every time, n (%)	Often, n (%)	Rarely, n (%)	Never, n (%)
Traveler’s diarrhea	125 (37.8)	134 (40.5)	59 (17.8)	13 (3.9)
Insect bite avoidance	127 (38.5)	130 (39.4)	55 (16.7)	18 (5.5)
In-flight exercise to prevent DVT	120 (36.5)	128 (38.9)	58 (17.6)	23 (7.0)
Infection outbreaks at destination	66 (20.1)	120 (36.6)	95 (29.0)	47 (14.3)
Motion sickness	78 (23.6)	120 (36.4)	89 (27.0)	43 (13.0)
Safe water and food	169 (51.1)	112 (33.8)	36 (10.9)	14 (4.2)
Animal bite avoidance	105 (32.0)	111 (33.8)	80 (24.4)	32 (9.8)
Personal safety (e.g. violence, accidents)	77 (23.5)	110 (33.5)	91 (27.7)	50 (15.2)
Finding medical assistance while abroad	60 (18.2)	110 (33.4)	90 (27.4)	69 (21.0)
Risk and prevention of STIs	62 (19.0)	106 (32.4)	96 (29.4)	63 (19.3)
First aid knowledge	58 (17.6)	95 (28.9)	105 (31.9)	71 (21.6)
Jet lag	64 (19.5)	92 (28.0)	103 (31.3)	70 (21.3)
Travel health insurance	43 (13.1)	78 (23.8)	102 (31.1)	105 (32.0)

DVT: Deep vein thrombosis, STIs: Sexually transmitted infections.

Table 3 describes the frequency of counseling travelers according to their destinations about main travel vaccines and malaria chemoprophylaxis. Among PCPs who offered pretravel consultations, the more frequently reported advice given was for seasonal flu vaccine (70.0% every time and 21.2% often), hepatitis A vaccine (57.6% every time and 29.7% often), hepatitis B vaccine (47.6% every time and 33.8% often), and malaria chemoprophylaxis (50.6% every time and 30.8% often). On the other hand, the less frequently reported advice given was for pre-exposure prophylaxis of rabies (21.1% every time and 35.5% often), and poliomyelitis vaccine (28.4% every time and 35.8% often).

**Table 3:** Distribution of counseling travelers (according to specific destinations) about travel vaccines and malaria chemoprophylaxis in the preceding 6 months in Qatar, 2020 (N = 332).

Variable	Every time, n (%)	Often, n (%)	Rarely, n (%)	Never, n (%)
Yellow fever vaccine	109 (33.0)	121 (36.7)	72 (21.8)	28 (8.5)
Poliomyelitis vaccine	93 (28.4)	117 (35.8)	75 (22.9)	42 (12.8)
Rabies (preexposure prophylaxis)	69 (21.1)	116 (35.5)	88 (26.9)	54 (16.5)
Meningococcal vaccine	148 (45.0)	116 (35.3)	51 (15.5)	14 (4.3)
Hepatitis B vaccine	156 (47.6)	111 (33.8)	47 (14.3)	14 (4.3)
Typhoid fever vaccine	160 (48.6)	103 (31.3)	42 (12.8)	24 (7.3)
Malaria chemoprophylaxis	166 (50.6)	101 (30.8)	46 (14.0)	15 (4.6)
Hepatitis A vaccine	190 (57.6)	98 (29.7)	31 (9.4)	11 (3.3)
Seasonal flu vaccine	231 (70.0)	70 (21.2)	21 (6.4)	8 (2.4)

### 3.3 Counseling of specific patient groups

**Fig. 1:**
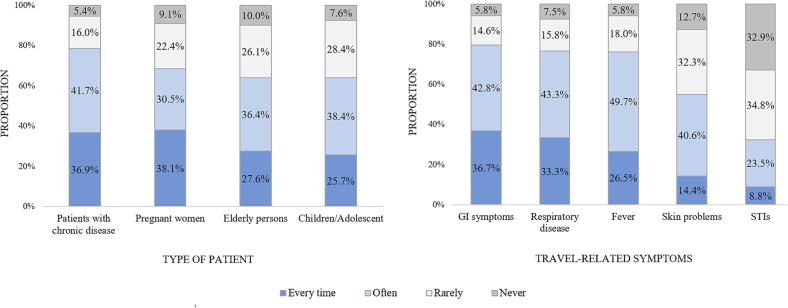
Distribution of counseling certain type of patient and travel-related symptoms.

Fig. 1 (left side) shows the distribution of counseling certain type of patient. Among PCPs who provided pretravel consultations, a high proportion provided pretravel consultations to patients with chronic diseases (36.9% every time and 41.7% often) and pregnant women (38.1% every time and 30.5% often). On the other hand, a relatively few PCPs counseled children/ adolescents or elderly persons.

### 3.4 Travel-related illnesses in returned travelers

Fig. 1 (right side) shows the distribution of travel-related illnesses in returned travelers. More than three-quarters of PCPs reported more frequent encounters with gastrointestinal (GI) symptoms, such as travelers’ diarrhea (36.7% every time and 42.8% often), respiratory diseases (33.3% every time and 43.3% often), or fever. On the other hand, STIs were the least encountered presentations.

### 3.5 Predictors of frequency of pretravel consultations

Table 4 displays the results of univariable and multivariable logistic regression analyses. In the univariable analysis, various PCP characteristics were significantly associated with a higher frequency of pretravel consultations (≥10/month). These variables included male gender, having 15 years or more in general practice, speaking ≥ 3 languages with patients, and having postgraduate training or experience in TM. The unadjusted OR and 95% CI for these variables are shown in Table 4.

**Table 4:** Predictors of providing ten or more travel medicine consultations per month among primary care physicians in Qatar, 2020 (N = 332).

Variable	Unadjusted OR (95% CI)	*p-value*	Adjusted OR (95% CI)	*p-value*
Age				
<45 years old	Reference		–	–
≥45 years old	1.11 (0.66, 1.88)	0.688	–	–
Gender				
Female	Reference		Reference	
Male	2.14 (1.24, 3.67)	<0.01*	1.78 (1.01, 3.18)	0.049*
Number of years in practice				
<15 years	Reference		Reference	
≥15 years	1.74 (1.03, 2.96)	0.039*	1.42 (0.81, 2.49)	0.223
Number of spoken languages with patients				
1-2 languages	Reference		Reference	
≥3 languages	1.77 (1.06, 2.96)	0.029*	1.96 (1.12, 3.45)	0.019*
Previous training or experience in TM^a^				
No	Reference		Reference	
Yes	2.36 (1.42, 3.92)	<0.001*	2.74 (1.59, 4.72)	<0.001*

* Statistically significant at a two-sided p<=0.05; OR: odds ratio, TM: travel medicine; CI: confidence intervals.

a Postgraduate experience in tropical medicine, developing countries, or communicable disease clinic.

In the multivariable analysis, male PCPs were more likely to provide pretravel consultation to travelers when compared to their female counterparts (AOR 1.78, 95% CI: 1.01, 3.18). Moreover, multilingual physicians were about twice as likely to counsel ten or more patients per month on TM matters compared to those who spoke fewer than three languages (AOR 1.96, 95% CI: 1.12, 3.45). Additionally, PCPs with postgraduate training or experience in TM were about three times more likely to counsel a higher frequency of patients (ten or more per month) on TM-related consultations compared to PCPs without such training or experience (AOR 2.74, 95% CI: 1.59, 4.72).

## 4 Discussion

This cross-sectional study aimed to examine TM practices among PCPs working in primary healthcare settings in Qatar, and the predictors of providing a higher frequency of pretravel consultations (≥10 per month). The current study showed that most PCPs (91.1%) provided pretravel consultations to patients, which is consistent with other research conducted in the United States of America (USA) (73%), Germany (95%), and Switzerland (96%) (Kogelman et al., 2014, Ropers et al., 2004, Hatz et al., 1997). In contrast, a study in Oman reported a relatively lower proportion of PCPs (58%) providing pretravel consultation (Kurup et al., 2019).

### 4.1 Pretravel advice: Frequency

Regarding the frequency of pretravel consultations, about three-quarters of the PCPs in the present study counseled less than 10 patients per month on TM issues. This is consistent with the findings of Kogelman and colleagues in the USA where the majority of the PCPs (80%) had counseled less than 10 patients each month on TM issues (Kogelman et al., 2014). In comparison, studies in Germany and Australia reported that the majority of PCPs counseled greater than 10 patients per month on TM-related issues (Ropers et al., 2004, Thava Seelan and Leggat, 2003).

Our regression analysis showed several predictors of the high frequency of consultations. One predictor was the multilinguality of the PCPs. Multilingual physicians were significantly more likely to consult a higher number of patients on travel-related issues than those who spoke only one or two languages. A similar finding was reported in a study conducted by Heywood and colleagues (2015) in Australia (Heywood et al., 2015). The ability to consult with patients in their preferred language potentially improves communication and trust and promotes change in their travel-related health behaviours (Gherardin, 2007). Such an ability is particularly important in Qatar where the population is highly diverse (Census Results, 2020).

Another predictor of higher frequency of pretravel consultations was PCPs’ postgraduate training or experience in TM. This is consistent with the findings from the studies in Germany and New Zealand (Ropers et al., 2004, Leggat et al., 1999). These findings emphasize the importance of training and experience in TM or a related field on the frequency of PCPs’ practice in TM consultations.

### 4.2 Pretravel advice: Content and frequency

There have been few studies investigating the nature of advice given by PCPs to travelers and how frequently such advice is provided. In the present study, advice on the prevention and management of travelers’ diarrhea (TD) was given by PCPs every time (38%) and often (41%). In contrast, 85% of PCPs in Germany (Ropers et al., 2004) and 78% in Oman (Kurup et al., 2019) had counseled travelers about TD. Advice regarding TD should be provided to all travelers regardless of their destination because it is the most common cause of morbidity in travelers (Bradley, 2017).

Another important topic of pretravel consultation is insect bite avoidance and its associated hazards of malaria infection in certain travel destinations. In this study, such advice was provided by PCPs every time (38%) and often (39%). Other studies in Germany, Oman, and Australia reported comparable findings (Ropers et al., 2004, Kurup et al., 2019, Thava Seelan and Leggat, 2003). It is vitally important that PCPs advise travelers going to malaria-endemic areas about the risks associated with insect bites. An earlier study in Qatar had shown that imported malaria had become increasingly a travel-related hazard from 2008 to 2015 (Farag et al., 2018).

On the subject of personal safety, such as violence, injuries, and accidents, only a small proportion of the PCPs provided advice against such events (23% every time and 33% often). Similar findings had been reported in Australia (Thava Seelan and Leggat, 2003). Safety is often a neglected area in TM, but it is one of the most important areas to cover when advising travelers because injuries and accidents are the most common preventable causes of death among them (World Health Organization. International travel and health., 2012). Finally; STIs are another important element in pretravel consultations (World Health Organization. International travel and health, 2012, Roupa et al., 2012). Despite this, only 19% of PCPs in the present study provided pretravel advice on the risk and prevention of STIs (every time), which is inconsistent with those found in studies in Germany (43%) and Australia (30%) (Ropers et al., 2004, Thava Seelan and Leggat, 2003). This could be explained by the sensitivity of the subject of STIs and its associated stigma in a conservative Muslim culture in this region such as Qatar.

### 4.3 Pretravel advice: Vaccines and malaria chemoprophylaxis

Overall, there is scarce published data regarding the provision of advice on specific vaccine recommendations. In the present study, we found that a relatively high proportion of PCPs provided advice on vaccinations against seasonal flu (70% every time and 21.2% often), hepatitis A (57.6% every time and 29.7% often), hepatitis B (47.6% every time and 33.8% often), meningococcal (45.0% every time and 35.3% often) and typhoid fever (48.6% every time and 31.3% often). Comparable rates of advice regarding hepatitis A and B were reported in other studies (Kogelman et al., 2014, Ropers et al., 2004, Hatz et al., 1997). Similarly, most PCPs in the current study provided advice on malaria chemoprophylaxis which is in line with findings in other international studies (Ropers et al., 2004, Thava Seelan and Leggat, 2003). However; only about a third of PCPs advised about yellow fever vaccine, which is also in line with other studies in the USA and Germany (Kogelman et al., 2014, Ropers et al., 2004). These findings are alarming because of the high risk of mortality associated with yellow fever. This study reported variations in PCPs’ practice of providing advice about specific vaccines. Therefore, PCPs should be encouraged to advise travelers about relevant vaccines depending on planned destinations.

### 4.4 Counseling specific patient groups

Many PCPs in our study provided pretravel advice to patients with chronic diseases (79%), pregnant women (69%), elderly persons (64%), and children/adolescents (64%). These findings are in line with the study in Germany (Ropers et al., 2004). Counseling these vulnerable groups plays an important role in primary care as they could be at a higher risk of acquiring travel-related illness or have contraindications to certain vaccines or medications (World Health Organization. International travel and health, 2012, Starr and Hagmann, 2020).

### 4.5 Post-travel consultations

In the present study, GI symptoms (e.g., TD) were the most frequent illness (80%) encountered by PCPs among returning travelers. Comparable findings were observed in the study in Germany (88%) and the study in Oman (77%) (Ropers et al., 2004, Kurup et al., 2019). This is an expected finding knowing that TD is the most predictable travel-related illness (World Health Organization. International travel and health ., 2012). Respiratory and skin diseases were other common presentations during post-travel consultation in this study in contrast to those reported in the German and Omani studies (Ropers et al., 2004, Kurup et al., 2019). These differences could be explained by the difference in travel destinations and the different characteristics and behaviours of travelers in different countries. In addition, few PCPs in this study encountered STIs among returning travelers who were ill. This finding suggests that PCPs should take detailed exposure histories and keep STIs in their differential diagnosis for such travelers.

### 4.6 Strengths and limitations

This study has a number of strengths. First, the study was the first of its kind to evaluate TM practice and its predictors among PCPs working in primary healthcare settings in Qatar. Second, the study achieved a high response rate (89.2%) despite the high demands placed on PCPs during the COVID-19 pandemic which could be explained by the PCPs’ interest in this topic. Lastly, the study includes PCPs' responses from all 27 PHC centers in Qatar, enhancing the generalizability of the findings to the broader scope of general practice in the country.

However, the cross-sectional design of this study limits its interpretation of the temporal relationship between the variables. Also, we acknowledge that the lack of precise data on patient volumes and the number of pretravel consultations limit our ability to analyze the relationship between these variables and provide further support for our inferences made. In addition, our study did not collect information on travel destinations, patient nationality, or the type of traveler, which could impact the advice provided by PCPs regarding travel-related illnesses. Future studies that incorporate such information could provide valuable insights for the development of travel medicine policies.

## 5 Conclusion

The majority of PCPs provided pretravel consultations. However, the frequency of consultations was mostly low. Male sex, past training or experience in TM and being a multilingual physician were important predictors of the provision of high frequency of pretravel consultations. The content and frequency of pretravel advice, including vaccine and malaria chemoprophylaxis recommendations, were inadequate. Travelers’ diarrhea, respiratory problems and fever were predominant travel-related issues seen by PCPs. In addition, a high proportion of PCPs provided pretravel consultations to patients with chronic diseases and pregnant women.

The findings of this study identified several gaps in PCPs’ TM practice. These potentially place travelers at high risk of acquiring TM-related diseases. Our findings will inform policy makers to design and implement specific measures, such as training and best practice guidelines for PCPs to standardize TM-related practice. Moreover, considering the culturally diverse population in Qatar, the use of professional medical interpreters when providing pretravel consultations and multilanguage information materials should be encouraged to overcome language barriers and enable effective counseling. Such measures may potentially promote healthy travel and reduce the burden of travel-related illnesses in Qatar.

## CRediT authorship contribution statement

**Ayman Al-Dahshan:** Conceptualization, Methodology, Formal analysis, Funding acquisition, Project administration, Writing – original draft. **Nagah Selim:** Writing – review & editing, Supervision. **Noora Al-Kubaisi:** . **Ziyad Mahfoud:** Formal analysis. **Vahe Kehyayan:** Writing – review & editing, Supervision.

## Declaration of Competing Interest

The authors declare that they have no known competing financial interests or personal relationships that could have appeared to influence the work reported in this paper.

## Data Availability

The datasets are available from the corresponding author on reasonable request.
