# Supplementary material for: Scope and Predictors of Travel Medicine Practice among Primary Care Physicians in Qatar
Source: Prev Med Rep. 2023 Jul 22;35:102337. doi: 10.1016/j.pmedr.2023.102337 (PMC10410236; doi:10.1016/j.pmedr.2023.102337)
Supplement: Supplementary data 1 [file mmc1.pdf]

Study Questionnaire

"National survey about practices of travel medicine and their associated factors among primary care physicians in Qatar, 2020"

Background characteristics

#	Question	Responses
1	**Age**	❑ (In completed years) ____________________
2	**Gender**	❑ Male ❑ Female
3	**Nationality**	❑ ____________________________________
4	**Native language**	❑ English ❑ Hindi<br>❑ Arabic ❑ Other, please specify<br>❑ Urdu ___________________
5	**Language(s) spoken with patients**<br>(select all applicable)	❑ English ❑ Hindi<br>❑ Arabic ❑ Others, please specify<br>❑ Urdu ___________________
6	**Total number of years in general practice**	❑ (In completed years) ____________________
7	**Number of all patients seen in your current general clinic per DAY**	❑ < 10 ❑ 20 – 29<br>❑ 10 - 19 ❑ ≥ 30
8	**Do you have previous postgraduate experience in tropical medicine/ developing countries?**	❑ No<br>❑ Yes (if yes, for how long?) …………………year(s)
9	**Have you ever run the CDC or travel clinic as part of your duties at PHCC?**	❑ No<br>❑ Yes
10	**Do you have previous postgraduate training in travel medicine or related area such as tropical medicine?**<br>*e.g.: postgraduate degree [Diploma, Master, PhD] or training [workshop, certified short course] or having a membership or fellowship of TM related professional organization*	❑ No<br>❑ Yes

Scope of practice in travel medicine

11	**What is the frequency of pretravel consultation in your current practice per MONTH?**	❑ I didn't provide pretravel consultations<br>❑ < 10 consultations<br>❑ 10-19 consultations<br>❑ ≥20 consultations
12	**What is the average duration of pretravel consultation** (counselling and prescription)?	❑ I didn't provide pretravel consultations<br>❑ < 10 minutes<br>❑ 10 - 19 minutes<br>❑ ≥20 minutes

A. "According to travel destination and based on up-to-date international recommendations such as CDC Travelers' Health, how frequently, in the past 6 months, did you provide counseling to travelers regarding the following pre-travel advice items?"

		Every time	Often	Rarely	Never
13	**Insect bite avoidance**	❑	❑	❑	❑
14	**Traveler's diarrhoea**	❑	❑	❑	❑
15	**Safe water and food**	❑	❑	❑	❑
16	**Animal bite avoidance**	❑	❑	❑	❑
17	**Risk and prevention of STIs**	❑	❑	❑	❑
18	**Personal safety** (e.g. violence, trauma or accidents)	❑	❑	❑	❑
19	**Infection outbreaks at destination**	❑	❑	❑	❑
20	**In-flight exercise to prevent DVT**	❑	❑	❑	❑
21	**Motion sickness**	❑	❑	❑	❑
22	**Jet lag**	❑	❑	❑	❑
23	**First aid knowledge**	❑	❑	❑	❑
24	**Finding medical assistance while abroad**	❑	❑	❑	❑
25	**Travel health insurance**	❑	❑	❑	❑

		Every time	Often	Rarely	Never
**B. "According to travel destination and based on up-to-date international recommendations such as CDC Travelers' Health, how frequently, in the past 6 months, did you provide counseling to travelers regarding the following pre-travel advice on travel vaccine or malaria chemoprophylaxis?"**					
**26**	**Malaria chemoprophylaxis**	❑	❑	❑	❑
**27**	**Seasonal flu vaccine**	❑	❑	❑	❑
**28**	**Hepatitis A vaccine**	❑	❑	❑	❑
**29**	**Hepatitis B vaccine**	❑	❑	❑	❑
**30**	**Typhoid fever vaccine**	❑	❑	❑	❑
**31**	**Yellow fever vaccine**	❑	❑	❑	❑
**32**	**Meningococcal vaccine**	❑	❑	❑	❑
**33**	**Poliomyelitis vaccine**	❑	❑	❑	❑
**34**	**Rabies** (Pre-exposure prophylaxis)	❑	❑	❑	❑
**C. In the past 6 months, how often did you provide the following patient groups with pretravel consultation?**					
		**Every time**	**Often**	**Rarely**	**Never**
**35**	**The elderly** (those who are $\geq$ 60 years old)	❑	❑	❑	❑
**36**	**Patients with chronic diseases**	❑	❑	❑	❑
**37**	**Pregnant women**	❑	❑	❑	❑
**38**	**Children/adolescents**	❑	❑	❑	❑
**D. In the past 6 months, how often did you encounter the following post-travel illness /presentation?**					
		**Every time**	**Often**	**Rarely**	**Never**
**39**	**GI symptoms** (e.g., travelers' diarrhea)	❑	❑	❑	❑
**40**	**Fever**	❑	❑	❑	❑
**41**	**Respiratory diseases**	❑	❑	❑	❑
**42**	**Skin problems**	❑	❑	❑	❑
**43**	**Sexual Transmitted Infections** (STIs)	❑	❑	❑	❑

Thank you for your participation in this study
